# UCB HCT in FLT3+ AML

**DOI:** 10.18632/oncotarget.21048

**Published:** 2017-09-19

**Authors:** Celalettin Ustun, Annalisa Ruggeri, Daniel J. Weisdorf

**Affiliations:** Celalettin Ustun: Division of Hematology, Oncology and Transplantation, Department of Medicine, University of Minnesota, Minneapolis, MN, USA

**Keywords:** AML, FLT3, umbilical cord blood, relapse, hematopoietic cell transplantation

Most common mutations in acute myeloid leukemia (AML) involving the *FLT3* gene (FLT3+) are either internal tandem duplication (FLT3-ITD, 15% to 35%) or missense point mutations (5% to 10%) in the tyrosine kinase (TK) domain (TKD) [1]. FLT3+ AML has been recognized as a special subset with clinical characteristics (e.g., in younger patients, presenting with higher white blood cell count, and poorer prognosis because of high relapse) [1]. New targeted therapies (e.g., tyrosine kinase inhibitors including midostaurin, gilteritinib, sorafenib) are highly effective and potentially improve survival in newly diagnosed or relapsed FLT3+ AML patients [2, 3]. Despite these recent improvements, allogeneic hematopoietic cell transplantation (HCT) is still considered as the most active consolidation therapy for cure of these patients [4].

Umbilical cord blood (UCB) HCT, as well as haploidentical HCT, has become an alternative option for patients in need of allogeneic HCT, but having no available HLA-matched siblings (MRD) or unrelated donors (MUD) [5, 6].

Relapse rates remain high, even after allogeneic HCT for patients with FLT3+ AML [7]. Therefore, in a large study including data from 2 registries [the Center for International Blood and Marrow Transplant Research (CIBMTR), and Eurocord and the European Society for Blood and Marrow Transplantation (EBMT)], we evaluated and compared UCB HCT (*n* = 126; single unit UCB HCTs, *n* = 50 and double units UCB HCTs, *n* = 76) outcomes with that of MRD (*n* = 67) and MUD (*n* = 91) HCTs in patients with FLT3+ AML [8]. The study’s focus was relapse after HCT. Relapse rates at 3 years were 44%, 33%, and 33% after MRD, MUD, and UCB HCTs, respectively. The relapse risk with UCB grafts was similar when compared with MRD (HR 0.86, 95%CI 052-1.42, *p* = 0.54) or with MUD (HR 1.05, 95% CI 0.65 - 1.69, *p* = 0.84). Non-relapse mortality (NRM) was higher with UCB HCT (HR 2.83, 95% CI 1.33 - 6.04, *p* = 0.007) compared with MRD HCT, but only marginally higher than MUD (HR 1.72, 95% CI 0.95-3.12, *p* = 0.07). Adjusted leukemia-free survival (LFS) at 3-years after MRD, UCB, and MUD HCTs was 43%, 39% and 50% (*p* = 0.42) while adjusted 3 year overall survival (OS) was 46%, 43% and 50%, respectively (*p* = 0.26). Compared with MRD, the HR after UCB HCT for LFS was 1.19 (95% CI 0.79 - 1.8, *p* = 0.4) and for OS was 1.36 (95% CI 0.9 - 2.06, *p* = 0.14). Again, UCB HCT and MUD HCT led to similar LFS and OS.

It is arguable that this combined registry study might be somewhat limited by the lack of data on the *FLT3* allelic burden and type of *FLT3* mutation (e.g., ITD or TKD). However, despite these limitations, this large study showed that UCB HCT outcomes were comparable with MRD and MUD in patients with FLT3+ AML. Added chemotherapy administered prior to HCT, necessitated by delays in MUD identification may result in increased FLT3 ligand plasma levels and thus resistance to targeted therapies. Additionally, because FLT3+ AML has high relapse rates, and that UCB grafts are readily available for many patients, UCB should be considered as a reasonable alternative for patients with FLT3+ AML to promptly facilitate allogeneic HCT.

Today approximately 40% of patients relapse after allogeneic HCT, regardless of donor source. This suggests that the graft-versus-leukemia effect along with the high dose chemoradiation conditioning regimen is inadequate for extended disease control posing the question whether use of an FLT3 active drug as maintenance after allogeneic HCT favorably influence the currently unacceptable high relapse rates? This intriguing question and the others including: the role of minimal residual disease at HCT, the impact of regimen intensity, the safety and influence of post HCT maintenance therapy on GVHD and cytopenias will be addressed in multicenter prospective studies such as the upcoming Blood and Marrow Transplant Clinical Trials Network (BMT CTN) prospective study randomizing patients to receive placebo vs. gilteritinib maintenance.

## References

[1] Pratz KW (2017). Blood.

[2] Stone RM (2017). N Engl J Med.

[3] Perl AE (2017). Lancet Oncol.

[4] Ho AD (2016). Biol Blood Marrow Transplant.

[5] Brunstein CG (2010). Blood.

[6] Ruggeri A (2010). Best Pract Res Clin Haematol.

[7] Gale RE (2005). Blood.

[8] Ustun C (2017). Leukemia.

